# Auditory Brainstem Response as a Diagnostic Tool for Patients Suffering From Schizophrenia, Attention Deficit Hyperactivity Disorder, and Bipolar Disorder: Protocol

**DOI:** 10.2196/resprot.3880

**Published:** 2015-02-12

**Authors:** Viktor Wahlström, Fredrik Åhlander, Rolf Wynn

**Affiliations:** ^1^Division of Addictions and Specialized PsychiatryUniversity Hospital of North NorwayTromsøNorway; ^2^Clinical Memory Research UnitDepartment of Clinical SciencesLund UniversityMalmöSweden; ^3^Department of Clinical MedicineFaculty of Health SciencesUiT The Arctic University of NorwayTromsøNorway

**Keywords:** brainstem audiometry, diagnosis, schizophrenia, ADHD, bipolar disorder

## Abstract

**Background:**

Psychiatric disorders, such as schizophrenia, attention deficit hyperactivity disorder (ADHD), and bipolar disorder, may sometimes be difficult to diagnose. There is a great need for a valid and reliable diagnostic tool to aid clinicians in arriving at the diagnoses in a timely and accurate manner. Prior studies have suggested that patients suffering from schizophrenia and ADHD may process certain sound stimuli in the brainstem in an unusual manner. When these patient groups have been examined with the electrophysiological method of brainstem audiometry, some studies have found illness-specific aberrations. Such aberrations may also exist for patients suffering from bipolar disorder.

**Objective:**

In this study, we will examine whether the method of brainstem audiometry can be used as a diagnostic tool for patients suffering from schizophrenia, ADHD, and bipolar disorder.

**Methods:**

The method includes three steps: (1) auditory stimulation with specific sound stimuli, (2) simultaneous measurement of brainstem activity, and (3) automated interpretation of the resulting brain stem audiograms with data-based signal analysis. We will compare three groups of 12 individuals with confirmed diagnoses of schizophrenia, ADHD, or bipolar disorder with 12 healthy subjects under blinded conditions for a total of 48 participants. The extent to which the method can be used to reach the correct diagnosis will be investigated.

**Results:**

The project is now in a recruiting phase. When all patients and controls have been recruited and the measurements have been performed, the data will be analyzed according to a previously arranged algorithm. We expect the recruiting phase and measurements to be completed in early 2015, the analyses to be performed in mid-2015, and the results of the study to be published in early 2016.

**Conclusions:**

If the results support previous findings, this will lend strength to the idea that brainstem audiometry can offer objective diagnostic support for patients suffering from schizophrenia, ADHD, and bipolar disorder. A positive result from the study could imply that brainstem audiometry could become an important supportive tool for clinicians in their efforts to diagnose patients with these disorders in a timely and accurate manner.

**Trial Registration:**

ClinicalTrials.gov NCT01629355; https://clinicaltrials.gov/ct2/show/NCT01629355 (Archived by WebCite at http://www.webcitation.org/6VBfTwx5H).

##  Introduction

Psychiatric disorders, such as schizophrenia, attention deficit hyperactivity disorder (ADHD), and bipolar disorder, may sometimes be difficult to diagnose [1]. The diagnostic criteria are complex and the disorders may present themselves in a wide variety of manners in clinical practice. The diagnoses are made primarily by comparing symptoms to sets of diagnostic criteria, often with the help of structured interviews [2]. Clinicians may use somatic examinations, various biochemical tests, electroencephalography, radiological examinations, etc, as aids in the diagnostic process. The investigations preceding the setting of these diagnoses are often extensive and time-consuming. There is a need for a valid and reliable diagnostic tool that can help clinicians in their efforts to diagnose these disorders in a timely and accurate manner. A diagnostic tool that could simplify the diagnostic process could free up resources that could be used to examine and treat more patients.

The brainstem is a hub for traffic in the central nervous system, including auditory activity. Evoked response audiometry is an electrophysiological method that records electrical signals—referred to as evoked potentials—that occur in the auditory system following acoustic stimulation [3-8]. By varying different elements in the examination, such as the time span of the examination, the acoustic properties of stimulation, the electrode placement and design, the number of signals forming the mean value, and the electrical filtering, different potentials may be studied. Areas of testing may involve masking functions (filters), habituation, lateralization (lateral asymmetry), and topographic patterns (eg, comparison between the peripheral activity and the thalamus activity) [9].

The early responses are studied with brainstem audiometry, also referred to as auditory brainstem response (ABR). This examination procedure has been used for many years in audiology departments, mainly for establishing the hearing threshold of patients, for aiding in the diagnosis of sensorineural hearing loss, and in the diagnosis of lesions and tumors in the brainstem. In this study, we will be drawing on a method that represents a further development of the standard ABR, referred to as SensoDetect Brainstem Evoked Response Audiometry (SD-BERA). It uses a wider array of acoustic stimuli, including complex sounds such as masking noises. The method consists of three parts: (1) auditory stimulation with specially designed sounds, involving functional and dysfunctional neuronal networks, (2) simultaneous reading of activity in the brainstem via electrodes, and (3) automated interpretation of the measurements with computer-based signal analysis.

Prior studies have suggested that patients suffering from schizophrenia and ADHD may process certain sound stimuli in the brainstem in an unusual or aberrant manner [3-20]. Some studies have suggested that when these patient groups have been examined with brainstem audiometry, the resulting brainstem audiograms have displayed illness-specific aberrations [9,14,17,21]. These aberrations have also been noted in unpublished observations. Previous studies have aimed to associate details in the audiograms with specific anatomical structures. This will be one of the first systematic studies drawing on this method to include patients with bipolar disorder.

In this study, we will examine whether the method of high-resolution brainstem audiometry can be used as a diagnostic tool for patients suffering from schizophrenia, ADHD, and bipolar disorder. We will study the use of a short (25 to 30 minutes) examination procedure. We will test the following hypotheses: (1) patients with schizophrenia, ADHD, and bipolar disorder differ from healthy subjects with respect to brainstem response measured with brainstem audiometry, (2) the diagnoses mentioned above may be reached in a blinded study, and (3) the method has sufficient sensitivity and specificity to be applied as a tool for diagnostic support for these illnesses.

##  Methods

### Inclusion Criteria, Exclusion Criteria, and Recruitment

Patients aged 18 to 70 years that have received a best-practice diagnosis—according to the 10th revision of the International Classification of Diseases (ICD-10) criteria [1]—of schizophrenia (section F20), ADHD (section F90), or bipolar disorder (section F31), at least one year prior to enrollment, may be included in the study.

Patients who are incapable of giving consent, who suffer from serious hearing loss, who have serious ongoing alcohol abuse or drug abuse issues, who have diagnosed psychiatric comorbidity, or who have a history of brain injury following cranial trauma will be excluded.

The patients will be recruited from the psychiatric departments at the University Hospital of North Norway (UNN) and from the registry at the General Practitioner Office in Storsteinnes, Balsfjord, Norway. Healthy control subjects of matching gender and age will be recruited from the population of the district of Troms, primarily students and employees at the psychiatric departments at UNN and from the General Practitioner Office in Balsfjord, Norway.

Information regarding the patients’ diagnoses and medications will be collected from their medical records.

The study has been approved by the Regional Medical Ethics Committee for North Norway (REC North 2011/2149).

### Study Design

This is a blinded study with brainstem audiometry, with the purpose of evaluating the predictive value of the test. A total of 48 individuals will participate in the study—a patient group consisting of 36 individuals with the above diagnoses (ie, three groups of 12 patients with each diagnosis) and 12 healthy individuals will undergo measurements. The resulting data will be analyzed under blinded conditions, according to a previously arranged data algorithm. This clinical trial has been registered at ClinicalTrials.gov (Identifier NCT01629355).

### Measurements

**Figure 1:**
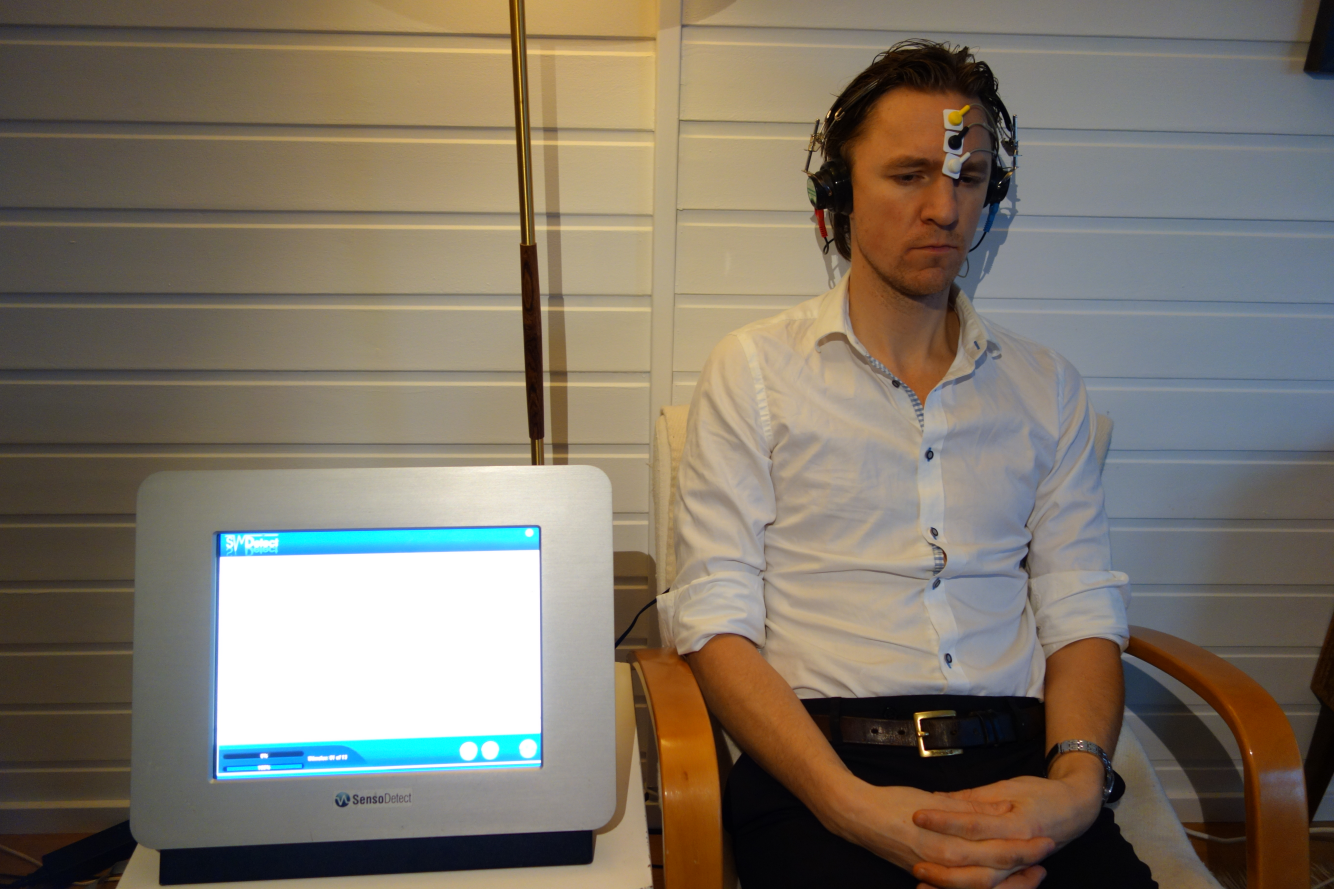
Demonstration of brainstem audiometry measurement equipment (first author pictured).

The actual measurement procedure is noninvasive. The examination takes 25 to 30 minutes and no preparation or active patient participation is required during the procedure. Five surface electrodes are attached to the skin on the forehead and behind the ears on the mastoid process. The examination involves the patient sitting comfortably reclined in an armchair in a quiet and dimmed room with headphones, listening to an array of acoustic stimuli not exceeding 70 dB (Figure 1). The method registers evoked potentials resulting from neural activity in the brainstem. These potentials occur within 10 msec following acoustic stimulation, are registered by the surface electrodes, and stored digitally. The method is European Conformity (CE) marked.

### Analysis

**Figure 2:**
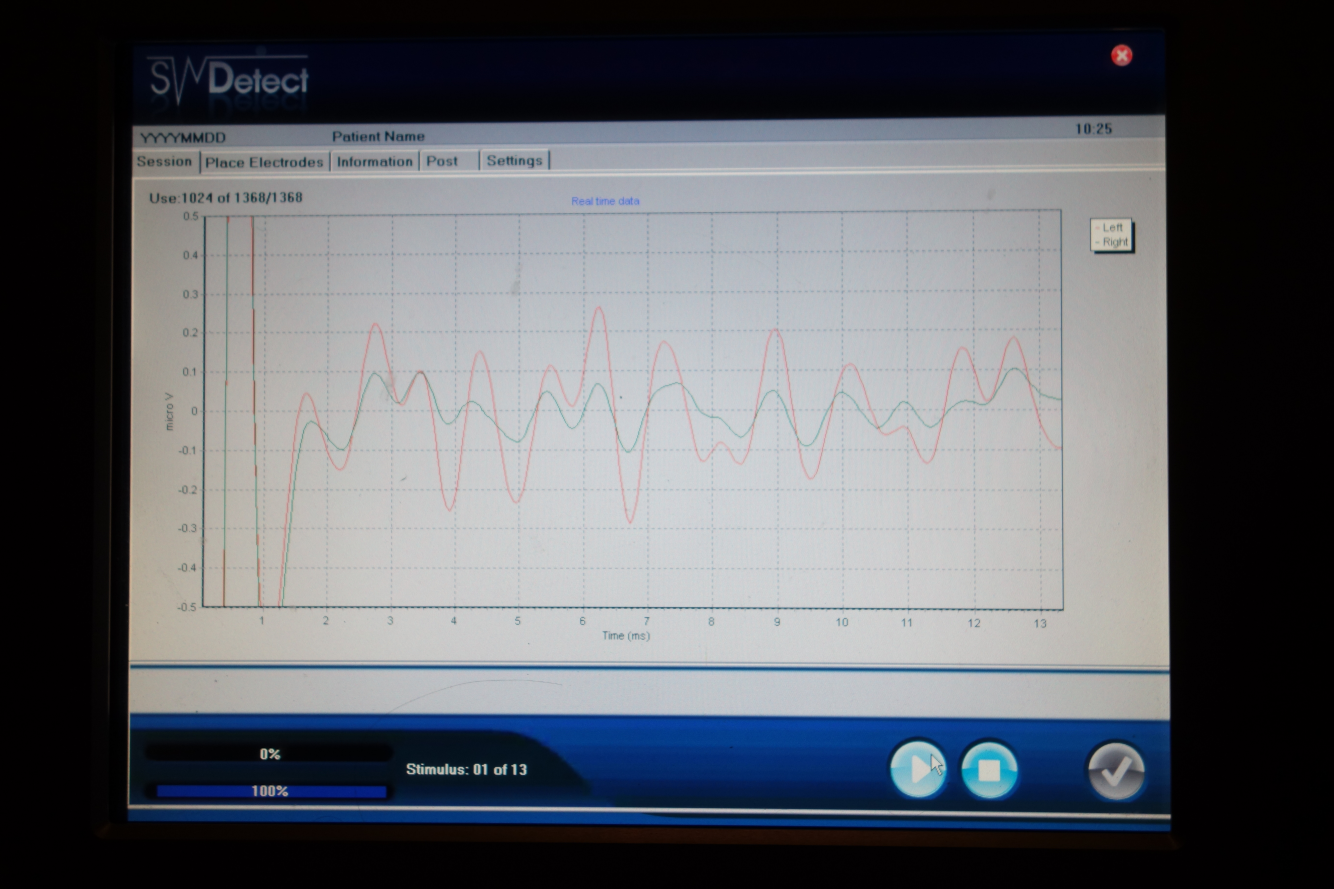
Illustrative photo of equipment used, demonstrating recorded waves.

The resulting wave pattern consists of seven peaks, which are interpreted with respect to latencies and amplitudes (Figure 2). From this pattern, abnormalities in the brainstem are detected and disease-specific patterns can be matched. A data algorithm is used in the analysis. In this study, the audiograms are anonymized and subsequently analyzed with paid assistance from SensoDetect, a company based in Lund, Sweden.

### Statistics and Power

Based on prior findings, we expect a sensitivity and specificity of 80%. A power calculation suggests that 12 patients in each patient category will be sufficient to detect statistical differences at the *P*<.05 level (ie, Fischer's exact test for the analysis of difference in the percentage of population response between groups, the Mann-Whitney test, or Wilcoxon test for the analysis of differences in values between groups).

##  Results

This project is now in a recruiting phase. When all patients and controls have been recruited and the measurements performed, the data will be analyzed according to a previously arranged algorithm. This analysis will be performed blindly by the company SensoDetect (ie, they will not know the diagnoses of the patients from which individual measurements were taken). We expect the recruiting phase and measurements to be completed in early 2015, the analyses to be performed in mid-2015, and the results of the study to be published in early 2016.

##  Discussion

The aim of this study is to evaluate whether the method is useful with respect to reaching a diagnosis of schizophrenia, ADHD, or bipolar disorder, and to assess, in a blinded study, the validity of the method for diagnosing these illnesses. The long-term benefit of this method could be to ensure a fast and accurate diagnosis for individuals suffering from schizophrenia, ADHD, or bipolar disorder, and thus be able to provide the patients with timely and adequate attention. Should the results confirm that the method represents a valid and reliable diagnostic support tool for these disorders, it may be significant to the field of psychiatry.

## Abbreviations

ADHD: attention deficit hyperactivity disorder
ABR: auditory brainstem response
CE: European Conformity
ICD-10: 10th revision of the International Classification of Diseases
SD-BERA: SensoDetect Brainstem Evoked Response Audiometry
UNN: the University Hospital of North Norway
